# Racial Disparities in Cardiovascular and Cerebrovascular Adverse Events in Patients with Non-Hodgkin Lymphoma: A Nationwide Analysis

**DOI:** 10.3390/medicina60050800

**Published:** 2024-05-11

**Authors:** Kanishka Uttam Chandani, Siddharth Pravin Agrawal, Maharshi Raval, Sajid Siddiq, Ahmed Nadeem, Ashish V. Chintakuntlawar, Shahrukh K. Hashmi

**Affiliations:** 1 Department of Internal Medicine, New York Medical College/Landmark Medical Center, Woonsocket, RI 02895, USA; kanishka.uttamchandani@gmail.com; 2 Department of Medicine, Smt. NHL Municipal Medical College, Ahmedabad 380006, Gujarat, India; 3 Department of Cardiology, New York Medical College/Landmark Medical Center, Woonsocket, RI 02895, USA; 4 Department of Hematology-Oncology, New York Medical College/Landmark Medical Center, Woonsocket, RI 02895, USA; 5 Division of Hematology and Oncology, Mayo Clinic, Phoenix, AZ 85054, USA; 6 Department of Medicine, Mayo Clinic, Rochester, MN 55905, USA; 7 College of Medicine and Health Sciences, Khalifa University, Abu Dhabi, United Arab Emirates; 8 Department of Computer Vision, MBZ University of Artificial Intelligence, Abu Dhabi, United Arab Emirates

**Keywords:** non-Hodgkin lymphoma, MACCE, racial disparities

## Abstract

*Background and Objectives*: Non-Hodgkin lymphoma (NHL) has the sixth-highest malignancy-related mortality in the United States (US). However, inequalities exist in access to advanced care in specific patient populations. We aim to study the racial disparities in major adverse cardiovascular and cerebrovascular events (MACCEs) in NHL patients. *Materials and Methods*: Using ICD-10 codes, patients with NHL were identified from the US National Inpatient Sample 2016–2019 database. Baseline characteristics, comorbidities, and MACCE outcomes were studied, and results were stratified based on the patient’s race. *Results*: Of the 777,740 patients with a diagnosis of NHL, 74.22% (577,215) were White, 9.15% (71,180) were Black, 9.39% (73,000) were Hispanic, 3.33% (25,935) were Asian/Pacific Islander, 0.36% (2855) were Native American, and 3.54% (27,555) belonged to other races. When compared to White patients, all-cause mortality (ACM) was significantly higher in Black patients (aOR 1.27, 95% CI 1.17–1.38, *p* < 0.001) and in Asian/Pacific Islander patients (aOR 1.27, 95% CI 1.12–1.45, *p* < 0.001). Sudden cardiac death was found to have a higher aOR in all racial sub-groups as compared to White patients; however, it was statistically significant in Black patients only (aOR 1.81, 95% CI 1.52–2.16, *p* < 0.001). Atrial fibrillation (AF) risk was significantly lower in patients who were Black, Hispanic, and of other races compared to White patients. Acute myocardial infarction (AMI) was noted to have a statistically significantly lower aOR in Black patients (0.70, 95% CI 0.60–0.81, *p* < 0.001), Hispanic patients (0.69, 95% CI 0.59–0.80, *p* < 0.001), and patients of other races (0.57, 95% CI 0.43–0.75, *p* < 0.001) as compared to White patients. *Conclusions*: Racial disparities are found in MACCEs among NHL patients, which is likely multifactorial, highlighting the need for healthcare strategies stratified by race to mitigate the increased risk of MACCEs. Further research involving possible epigenomic influences and social determinants of health contributing to poorer outcomes in Black and Asian/Pacific Islander patients with NHL is imperative.

## 1. Introduction

Non-Hodgkin lymphoma (NHL) accounts for about 90% of all lymphomas, while Hodgkin’s lymphoma forms 10% of the disease burden. Within its vast spectrum of B-cell, T-cell, and natural killer (NK) cell-related hematological malignancies, NHL includes approximately 60 different types, including some rare sub-types [1], with the most common subtypes of NHL being B-cell NHLs (75%), encompassing mainly diffuse large B-cell lymphoma (DLBCL) and follicular lymphoma (FL) [2]. In addition to being the most common sub-type of lymphomas, NHL is also the seventh-most prevalent cancer and has the sixth-highest malignancy-related mortality in the United States [3].

Advancements in the treatment approach for NHL, including the introduction of the R-CHOP regimen consisting of rituximab, a CD20 inhibitor, cyclophosphamide, doxorubicin, vincristine, and prednisone for B-cell NHLs; T-cell-directed therapies; radiation; and novel therapies, like hematopoietic stem cell transplant and chimeric antigen receptor T-cell (CAR-T), have led to a substantial improvement in the 5-year survival of patients with NHL in developed countries. The 5-year survival of patients with NHL who were diagnosed between 2012 and 2018 is currently somewhere between 64 and 86% in the United States, depending on the stage of NHL [4]. This is a remarkable improvement in the 5-year survival as compared to a mere 46.6% in 1975 [5]. However, as the 5-year survival rates improve, addressing major adverse cardiovascular and cerebrovascular events (MACCEs) becomes increasingly paramount. Age has been known to be a significant risk factor and poses a global health challenge in the pathogenesis of cardiovascular and, subsequently, cerebrovascular disease [6]. Considering the increasing age of NHL patients due to longer overall survival rates, the malignancy itself, as well as the effects of potentially cardiotoxic chemotherapy regimens used in its treatment, MACCEs become increasingly critical.

Despite these treatment advances over the past few decades, various studies have highlighted that racial and socioeconomic disparities do exist in the overall survival of patients with different hematological malignancies, including multiple myeloma, chronic lymphocytic leukemia (CLL), and follicular B-cell lymphoma, a sub-type of NHL. Furthermore, these disparities have been found to exist in NHL itself, affecting the prevalence and overall survival [7,8]. These discrepancies have been hypothesized to be related to differential occupational and environmental exposures, which may be significantly affected by different socioeconomic factors [9].

Extending this concept into the prevalence of MACCE outcomes in patients with NHL, this study aims to explore the racial disparities in MACCE outcomes in these patients. We hypothesize that inequalities may be present in the prevalence of MACCE outcomes in different races of patients with NHL, which may possibly be attributable to disparities in healthcare access, including but not limited to access to advanced services and clinical trials. This study aims to highlight the presence of critical healthcare gaps through an analysis of a national database. Understanding the presence of this gap could subsequently allow for advocacy for equitable healthcare access across all races in patients with NHL.

## 2. Methods

### 2.1. Study Design and Data Source

The National Inpatient Sample (NIS) database from 2016 to 2019 was used in this study. This database is a publicly available resource under the Healthcare and Utilization Project of the Agency for Healthcare Research and Quality (AHRQ) in the United States. Datasets from each year contain information from 20% of discharges from community hospitals (excluding rehabilitation and long-term acute care institutions). Further statistical analysis of these datasets provides national estimates of different variables, including inpatient hospitalizations, use, access, costs, demographic profiles, income profiles, and outcomes for the United States. Institutional Review Board approval was not required as the NIS contains de-identified data. A multivariate regression analysis was performed to reduce the potential for bias.

### 2.2. Study Population and Variables

ICD-10 codes were used to identify patients with various sub-types of NHL, including follicular lymphoma (C.82), non-follicular lymphoma (C.83), mature T/NK lymphomas (C.84), other specified and unspecified types of non-Hodgkin lymphoma (C.85), other specified types of T/NK-cell lymphoma (C.86), malignant immunoproliferative diseases and certain other B-cell lymphomas (C.88), and extranodal marginal zone B-cell lymphoma of mucosa-associated lymphoid disease (MALT-lymphoma) (C88.4).

All patients above the age of 18 years with a diagnosis of NHL and its subtypes were first identified using the ICD-10 codes mentioned before. Demographic data were extracted, including age, sex, insurance type, and median household income quartiles. Additionally, the prevalence of various cardiovascular comorbidities was identified. These cohorts were then further stratified by race to study the outcomes of interest, including the cardiovascular disease (CVD) burden, major adverse cardiovascular and cerebrovascular events (MACCEs), and healthcare resource use. The patient population whose data pertaining to race were not available was excluded from the study. Age in a continuous manner and other variables (race; admission type; hospital characteristics, including hospital bed size (small, medium, or large), location/teaching status (rural, urban non-teaching, or urban teaching), and region; median household income quartiles and payer status; disposition type; and death as an outcome) in a categorical manner were used for statistical analysis.

Baseline characteristics, including demographics, comorbidities, and MACCE outcomes, were studied using the chi-square test for categorical and ANOVA for continuous data (statistical significance determined as *p*-value < 0.05), and results were stratified based on patients’ races.

### 2.3. Outcomes

We included in-hospital all-cause mortality (ACM), acute myocardial infarction (AMI), atrial fibrillation (AF), cerebrovascular accident (CVA), and sudden cardiac death (SCD) as MACCEs for outcomes. ACM was available as the NIS has an in-built variable “died” corresponding to in-hospital deaths. All other outcomes were extracted from discharge diagnoses using ICD-10 codes. The NIS has an in-built variable “race”, with the following categories: White, Black, Hispanic, Asian/Pacific Islander, Native American, and other. MACCEs were compared among these races in patients diagnosed with NHL or any of its subtypes, as defined before.

### 2.4. Statistical Analyses

The analysis was carried out with STATA software, version 13.0 (created by StataCorp in College Station, TX, USA). The NIS uses a sophisticated sampling methodology that includes stratification, clustering, and weighting. This approach ensures that the analysis produces nationally representative and unbiased results, as well as variance estimates and *p*-values. Patient-level observations were weighted to extrapolate results to the entire hospitalized population with NHL in the United States. Discharge records with missing data for race (<5%) were excluded from the final analysis. Baseline characteristics, including demographics, comorbidities, and MACCE outcomes, were compared among races using the chi-square test (statistical significance determined as a two-sided *p*-value < 0.05) for categorical and the ANOVA test for continuous data. The adjusted odds ratios (aORs) of MACCEs were estimated, with Whites as the reference group, using multivariate logistic regression. Multivariate regression models were created by including all confounding factors that had a significant association with the outcome during univariable analysis, with a significance cutoff of 0.2. Furthermore, variables identified as critical determinants of outcomes based on the existing literature were included in the models regardless of their statistical significance in the univariate analysis.

### 2.5. Ethical Considerations

This study did not require approval from the Institutional Review Board (IRB) or informed consent as it was a retrospective study conducted using the National Inpatient Sample (NIS) database from 2016 to 2019, which contains de-identified data from 20% of all hospitalizations nationally in the United States. This study aims to highlight racial disparities in healthcare in order to advocate for a more equitable distribution of healthcare resources.

## 3. Results

**Figure 1:**
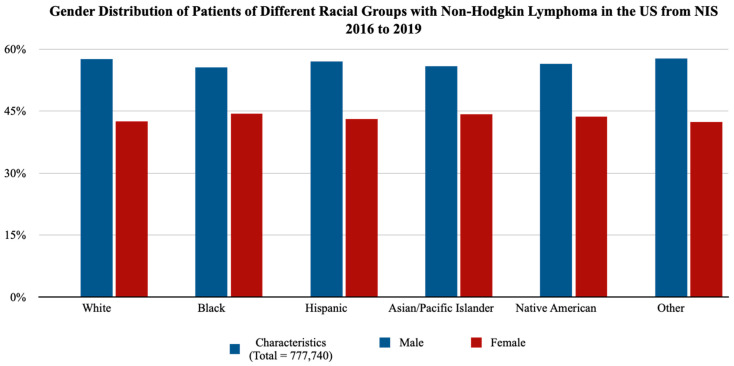
Gender distribution of patients of different racial groups with non-Hodgkin lymphoma in the United States from the NIS database from 2016 to 2019.

In total, 777,740 admitted patients with NHL were identified from the NIS database from 2016 to 2019 and included in the study, of which 56.66% were male and 43.33% were female (Figure 1). The majority of the hospitalized patients were White (74.22%, 577,215 patients) and Hispanic (9.39%, 73,000 patients), followed by Black (9.15%, 71,180 patients), other races (3.54%, 27,555 patients), Asians/Pacific Islanders (3.33%, 25,935 patients), and Native Americans (0.36%, 2855 patients). The mean age was the highest in White patients (67.75 years) and Native American patients (62.96 years), with Asian/Pacific Islander patients having a similar mean age of 62.87 years, followed by patients belonging to other races (61.08 years) and Hispanic patients (60.22 years). The lowest mean age was observed in Black patients (58.08 years). Simultaneously, the highest percentage of non-elective admissions was also noted in Black patients (79.11%), followed by Native American patients (77.15%), White patients (75.17%), and Hispanic patients (74.24%). The lowest percentage of non-elective admissions was observed in patients of other races (70.26%) and Asians/Pacific Islanders (70.16%). Upon analysis of income quartiles, it was observed that most of the patients who were Black (48.18%) and Native Americans (41.82%) belonged to the lowest income quartile, whereas 48.25% of the Asian/Pacific Islander patients were found to belong to the highest income quartile (Table 1).

Various comorbidities were assessed in patients with NHL and were further sub-categorized by race (Table 2). Hypertension was the most common comorbidity among all races, with the prevalence ranging from 34.35% to 36.75%. Diabetes mellitus was the most prevalent comorbidity in Native American patients (33.8%) and Hispanic patients (29.32%). Smoking was found to be a significant comorbidity in Native American and Black patients (12.43% and 10.64%, respectively). White and Asian/Pacific Islander patients were found to have the highest prevalence of dyslipidemia at 36.52% and 32.64%, respectively, versus patients who were Black (25.44%), Hispanic (27.29%), Native American (29.77%), and of other races (26.84%). Obesity was similarly prevalent in both Black (11.46%) and Native American (11.73%) patients. Some degree of renal failure was present in about a fourth of the Black patients (24.89%), as well as in patients who were White (20.45%), Hispanic (18%), Asian/Pacific Islander (17.22%), Native American (20.49%), and of other races (17.53%). Congestive heart failure was present across major racial groups in White (17.10%), Black (16.82%), and Native American (15.24%) patients. Chronic obstructive pulmonary disease was most prevalent in White (15.59%) and Native American (14.71%) patients. Other comorbidities, including pulmonary circulation disease and depression, were more common in Native American patients (7.88% and 12.78%, respectively), and coagulopathies were noted more commonly in Asian/Pacific Islander patients (1.39%).

Hospitalization charges in US dollars were found to be significantly higher in Asian/Pacific Islander patients (USD 114,352.80), Hispanic patients (USD 111,004.60), and patients of other races (USD 110,832.20) as compared to White patients (USD 81,533.52), with *p*-value <0.001. Concomitantly, the highest self-pay sub-groups were found to be patients of other races (5.17%) and Hispanic patients (5.12%) as compared to White patients (1.21%).

**Figure 2:**
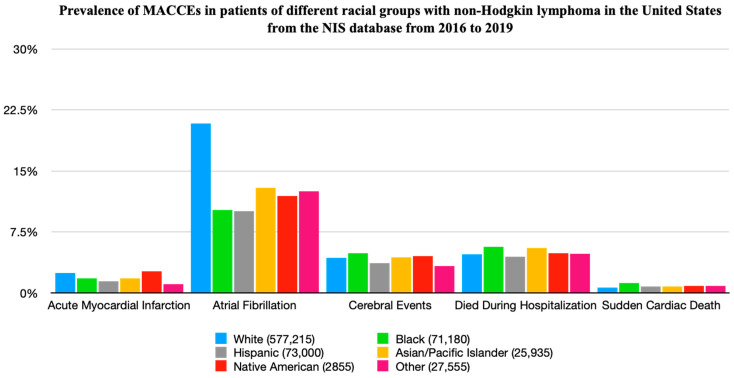
Prevalence of MACCEs in patients of different racial groups with non-Hodgkin lymphoma in the United States from the NIS database from 2016 to 2019.

The prevalence and adjusted odds ratios (aORs) of various MACCEs among racial sub-groups were analyzed (Table 3 and Table 4, Figure 2). The most common MACCE was atrial fibrillation, with the highest prevalence in White patients (20.84%), followed by Asian/Pacific Islander patients (12.92%) and patients of other races (12.52%). AMI was the highest in Native American patients (2.63%), followed by White patients (2.45%). CVA had the highest prevalence in Black patients (4.87%) and Native American patients (4.55%). The prevalence of SCD and death during hospitalization was the highest in Black patients at 1.22% and 5.64%, respectively. The aOR of ACM with White patients as a reference was significantly higher in both Black patients (aOR 1.27, 95% CI 1.17–1.38, *p* < 0.001) and Asian/Pacific Islander patients (aOR 1.27, 95% CI 1.12–1.45, *p* < 0.001), followed by patients of other races (aOR 1.18, 95% CI 1.01–1.38, *p* = 0.032). SCD was found to have a higher aOR in all racial sub-groups as compared to White patients; however, it was statistically significant in Black patients only (aOR 1.81, 95% CI 1.52–2.16, *p* < 0.001).

AF was noted to have a statistically significant lower aOR in all racial sub-groups as compared to White patients, with the odds being the lowest in Hispanic patients (aOR 0.57, 95% CI 0.53–0.61, *p* < 0.001), followed by similar ratios in other sub-groups, including Native American patients (aOR 0.61, 95% CI 0.44–0.83, *p* = 0.002), Black patients (aOR 0.61, 95% CI 0.57–0.65, *p* < 0.001), Asians/Pacific Islanders (aOR 0.69, 95% CI 0.62–0.77, *p* < 0.001), and other races (aOR 0.77, 95% CI 0.70–0.84, *p* < 0.001). Similarly, AMI was noted to have a statistically significant lower aOR in Black patients (0.70, 95% CI 0.60–0.81, *p* < 0.001), Hispanic patients (0.69, 95% CI 0.59–0.80, *p* < 0.001), and patients of other races (0.57, 95% CI 0.43–0.75, *p* < 0.001) as compared to White patients. However, CVA had no statistically significant aOR in the identified sub-groups.

## 4. Discussion

Using the NIS database, this study analyzed demographic patterns, healthcare resource use, comorbidities, and significant cardiovascular and cerebrovascular events in 777,740 hospitalized patients with NHL from 2016 to 2019. Overall, White and Hispanic patients formed the majority of the patient population, with patients who were Black, Asian/Pacific Islander, Native American, and of other races being in the minority. Notably, almost half of the Black and Native American patients belonged to the lowest income quartile. Upon analysis of comorbidities, hypertension prevailed as the most common comorbidity across patients of all races. On the contrary, other comorbidities showed racial variations in prevalence, with dyslipidemia, congestive heart failure (CHF), and chronic obstructive pulmonary disease (COPD) being most prevalent in White patients. Similarly, renal failure was found to affect about a fourth of the Black patients, and the Native American patients were found to have the highest prevalence of obesity, smoking, and diabetes mellitus. Significant racial differences were observed in Black patients, who were found to have the highest prevalence of cerebral events, SCD, and death during hospitalization. In contrast, AMI was more commonly seen in Native American patients, and AF was most prevalent in the White patient population. The aORs of ACM and SCD showed statistically significant racial disparities in racial minority groups. However, AMI and AF were found to affect White patients more, given an aOR of <1 in all other ethnic groups. Healthcare resource use was measured using median hospitalization charges, which were found to be the highest in patients who were Hispanic, Asian/Pacific Islander, and of other races.

Cardiovascular disease (CVD) and cancer are both known to be leading causes of mortality globally [10]. Interestingly, they share several associated factors spanning from diabetes mellitus and dyslipidemia to cachexia and an impaired immune response [11]. CVD and cancer are intertwined, and this relationship is further being studied in the fields of cardio-oncology and reverse cardio-oncology. A study conducted on mice found that early cardiac remodeling could potentially nurture tumor growth and metastasis and subsequently accentuate cancer progression [12]. A similar study in mice also revealed that the presence of heart failure resulted in a significantly increased intestinal tumor load of 2.4-fold in APC^min^ mice (*p* < 0.0001) [13]. However, it is also to be acknowledged that not all murine experimental findings may translate into humans, despite mice being used as the most common in vivo experimental animal, owing to 65 million years of evolutionary mutations [14].

Concurrently, the presence of cancer leads to worse cardiovascular outcomes due to both cancer progression and a consequence of cancer treatment, including both traditional chemotherapy and newer targeted therapy regimens [15,16]. It has been postulated and demonstrated that this association can be explained at a cellular level due to the presence of microRNAs. miR-22 is an oncomiR that is known to be a significant contributor in multiple cancers, including breast cancer, hepatocellular carcinoma, and acute myeloid leukemia (AML), while also being a key player in the development of cardiac fibrosis, thus strengthening this postulated connection [10]. Overall, a national study using the Surveillance, Epidemiology, and End Results (SEER) program established that CVD mortality risk was the highest, with a standardized mortality ratio of 3.93 (95% CI 3.89–3.97), within the first year of cancer diagnosis, and the CVD mortality risk was persistently elevated throughout the follow-up period, as compared to the general population [17].

Of the 777,740 patients included in our study, 2855 were Native American, and they were noted to have the highest prevalence of diabetes mellitus, smoking, obesity, depression, and pulmonary circulation disease, which likely translated into them having the highest prevalence of AMI at 2.63%. These data were comparable to a national database study using the Healthcare Cost and Utilization Project National Inpatient Sample from 2000 to 2018, which demonstrated that Native Americans were the second-highest population, only second to White patients, with high AMI rates, with the trend going upward from 14.0/1000 to 16.1/1000 over the course of 18 years [18]. Moreover, Native Americans have been found to have a disproportionately higher rate of premature myocardial infarction (female < 65 years, male < 55 years) as compared to all other racial groups combined, with the rate in Native Americans being 22.8% versus 14.8% in all other racial groups combined [19]. This may possibly be explained by the intricate interplay of cancer with CVD.

Furthermore, the Black patients included in our study exhibited a relatively higher prevalence of hypertension and renal failure. They were noted to have the highest prevalence of CVA, ACM, and SCD. Additionally, they were at a higher risk of ACM and SCD when compared explicitly to White patients. Concomitantly, various studies have outlined the disparities that exist in Black and Native American patients with respect to survival in NHL over the course of 40 years, with overall survival (OS) being poorer in these populations. Moreover, the difference in the OS from 1973 to 2011 in T-cell NHL was 3.1 years in White patients, while it was merely 1.7 years among Black patients (*p* < 0.001). Similarly, in B-cell DLBCL, the most common type of B-cell NHL, the OS was lower in Black patients (3.3 years) as compared to White patients (5.8 years), with *p* < 0.001 [20]. This may possibly be explained by the role of doxorubicin as one of the most active agents in the primary therapy for NHL, which is R-CHOP. Doxorubicin has been known for several decades for its late cardiotoxic effects in cancer survivors, especially those who receive over 400 mg per square meter of body surface area. Moreover, in the realm of the known cardiotoxicity of doxorubicin, Black patients have had a higher rate of this adverse effect, with an odds ratio of 2.93, than the overall population, possibly due to subtle genetic differences in different ethnicities [21], as being explored in the field of pharmacogenomics.

Hispanic patients formed 9.38% of the study population. They were noted to have a relatively high prevalence of hypertension, diabetes mellitus, and dyslipidemia (27.29%), as well as high healthcare use costs, with the mean hospitalization charges being USD 111,004.6. Our study highlighted that certain MACCEs, including AMI and AF, are less likely to occur in Hispanic patients as compared to White patients with NHL. On the contrary, a study using the SEER database noted that T/NK cell neoplasms are higher in the Hispanic population than in the non-Hispanic population (rate ratio 3.03, *p* = 0.0001) [22]. It has also been noted that Hispanic patients have poorer access to both traditional chemo- and/or immunotherapy as compared to White patients with an OR of 0.78 (95% CI 0.64–0.96) [23], as well as novel therapies for hematological malignancies, including CAR-T therapy [24]. An analysis of the NIS sample from 2018 observed that merely 13.3% of the overall patients receiving CAR-T therapy for NHL, acute lymphoblastic leukemia (ALL), and multiple myeloma were Hispanic and were observed to have poor access to centers offering CAR-T therapy [25]. Traditional chemoradiotherapy, immunotherapy, and CAR-T therapy are known to have significant cardiotoxic effects. Doxorubicin, one of many agents used to treat NHL, is known to be cardiotoxic [26]. Additionally, CAR-T therapy has been associated with cardiotoxic adverse effects, especially in populations with preexisting risk factors, like prior cardiac history and dyslipidemia, among others [27]. The most common adverse effects are mediated by cytokine release syndrome and are arrhythmias, heart failure, and sudden cardiac arrest [28]. Understanding that Hispanic patients have relatively poorer access to various treatment options for NHL is critical in explaining the lower odds ratio of various MACCEs, including AMI and AF.

Asian/Pacific Islander patients with NHL were observed to have the highest odds of ACM, with an aOR of 1.27 (95% CI 1.12–1.45, *p* < 0.001), as compared to White patients. This is in accordance with the known worst 5-year survival of DLBCL at 35% in Asian/Pacific Islander patients as compared to 41% in White patients [29]. The etiology, although not clearly defined at present, may come to light once more large-scale clinical trials with equitable racial representation are performed to delineate this association further.

This study, although a cross-sectional one, does shed light on the presence of racial disparities in MACCEs in patients admitted with a known diagnosis of NHL. With improving the overall survival of patients with NHL, the role of survivorship and gauging the long-term cardiovascular and cerebrovascular event risk becomes critical. Further prospective studies involving data from outpatient settings with NHL as the principal diagnosis and analyzing racial disparities in MACCE outcomes would help confirm the results of our study. Future research could potentially focus on the identification of various epigenomic and genetic factors that may lead to the development of this differential distribution of adverse events in various ethnic groups. Furthermore, the impact of the socioeconomic status could be explored as Black and Native American patients were found to be in the lowest income quartile and Black patients were found to be most affected by CVA, ACM, and SCD and Native American patients by AMI. Moreover, the emerging importance of the field of cardio-oncology is underscored, and it may be postulated that early involvement of a multidisciplinary team, including cardio-oncology, may lead to early diagnosis and intervention in these patients and subsequently translate into better outcomes. Ultimately, this study draws attention to the presence of critical healthcare gaps in people of color, which would require intervention with the development of more equitable healthcare, as well as preventive care.

### Limitations

The study has a few limitations, given that the NIS database permits only retrospective analysis of datasets. First, although ICD-10 codes were used, the possibility of inclusion and exclusion errors does exist in our cohort, owing to coding errors in this dataset. Sampling bias may have occurred as the NIS is a database of hospital discharges, and the overall generalizability of results may be limited due to this.

Second, it is to be noted that patients rarely get admitted with a primary diagnosis of NHL and may have some confounders. Outcomes noted in our study could be secondary to NHL, or they could be secondary to the primary condition for which the patient was admitted. However, this study, being a cross-sectional one, can only potentially shed light on associations but cannot establish causality. This would require prospective cohort studies, and this study can aid in highlighting the need to focus on MACCEs in clinical trials, while simultaneously ensuring adequate racial representation, which would further aid in understanding the finer nuances of various epigenomic and genetic factors that can cause significant differences in adverse events.

Third, the dataset is generated using codes that are primarily used for billing purposes, and thus, clinical data and diagnosis may be missed. Moreover, outpatient data are not available, and the data are visit based, which may lead to the same patient being counted multiple times.

Moreover, this study also would be unable to capture diagnoses of SCD made by emergency medical services (EMS), which may affect the results.

Additionally, this study identified patients with a variety of subtypes, ranging from indolent to more aggressive subtypes. It is imperative to recognize that various subtypes of NHL may behave differently and are treated with different types of chemotherapy regimens with diverse side effect profiles, although they come under the same umbrella of NHL. Further studies that stratify patients based on the nature of NHL would be required to understand the impact this may have on MACCEs.

Furthermore, we also did not have access to the medications, including specific chemotherapy regimens, that the patients may have been on, which can potentially cause MACCEs.

Despite these limitations, we provided contemporary data on cardiovascular and cerebrovascular events in patients with NHL from a large, nationally representative database, with detailed analysis.

## 5. Conclusions

Our study highlights the racial disparities in MACCE outcomes in patients with NHL, using the NIS database from 2016 to 2019. Black patients were noted to have a higher risk of ACM and SCD as compared to White patients. Similarly, Native Americans displayed the highest prevalence of CVA, ACM, and SCD. Conversely, Hispanic patients were noted to have lower odds of developing AF and AMI, and the disparities may influence access to both traditional and novel treatments for NHL. Asians/Pacific Islanders were noted to have the highest ACM. Thus, the importance of tackling racial disparities in the overall management of NHL, especially major cardiovascular and cerebrovascular events and outcomes, is underscored in this study. This may be achieved by emphasizing the need for equitable ethnic representation in clinical trials and by focusing on potential key risk factors, including but not limited to diet, smoking, and social-financial toxicities. This may help us better understand the phenotypic differences in the response to treatment and adverse effects due to genotypic variations. Further policy changes may also be warranted to allow for more tailored and equitable access to healthcare across all races.

## Figures and Tables

**Table 1:** Characteristics of patients of different racial groups with non-Hodgkin lymphoma in the United States from the NIS database from 2016 to 2019.

Characteristics (Total = 777,740)	White (577,215)	Black (71,180)	Hispanic (73,000)	Asian/Pacific Islander (25,935)	Native American (2855)	Other (27,555)	*p*-Value
**Characteristics**							
Male	57.55%	55.59%	56.95%	55.78%	56.39%	57.71%	0.01
Female	42.45%	44.41%	43.05%	44.22%	43.61%	42.29%	0.01
Age (years)	67.75418	58.08492	60.22411	62.87527	62.96844	61.08111	<0.0001
Non-elective admissions	75.17%	79.11%	74.24%	70.16%	77.15%	70.26%	<0.0001
**Primary expected payer (uniform)**							<0.0001
Medicare	63.40%	43.49%	41.80%	45.49%	55.01%	44.45%	
Medicaid	5.75%	21.78%	22.79%	14.14%	18.45%	12.81%	
Private insurances	27.31%	27.55%	26.75%	35.69%	21.44%	34.57%	
Self-pay	1.21%	3.74%	5.12%	2.41%	2.11%	5.17%	
No charges	0.10%	0.36%	0.95%	0.33%	0.00%	0.45%	
Others	2.24%	3.08%	2.58%	1.95%	2.99%	2.54%	
**Median household income national quartile for patient zip code (percentiles) ^#^**							<0.0001
0–25th	19.77%	48.18%	34.48%	10.49%	41.82%	22.31%	
26–50th	25.59%	21.48%	25.03%	15.72%	27.14%	19.88%	
51–75th	27.02%	18.29%	23.48%	25.54%	18.40%	25.06%	
76–100th	27.62%	12.05%	17.01%	48.25%	12.64%	32.76%	
**Bed size of the hospital ^§^**							<0.0001
Small	16.11%	13.84%	12.66%	11.20%	18.04%	9.69%	
Medium	25.07%	24.35%	25.17%	22.54%	19.09%	18.64%	
Large	58.82%	61.81%	62.16%	66.26%	62.87%	71.67%	
**Location/teaching status of the hospital ^~^**							<0.0001
Rural	6.32%	2.66%	1.24%	0.98%	11.73%	1.14%	
Urban non-teaching	16.81%	11.46%	15.68%	13.50%	12.96%	10.36%	
Urban teaching	76.86%	85.89%	83.08%	85.52%	75.31%	88.50%	
**Region of hospital**							<0.0001
Northeast	22.98%	18.88%	17.27%	19.39%	9.28%	27.36%	
Midwest	25.95%	19.08%	8.08%	9.22%	17.16%	7.95%	
South	33.12%	52.94%	37.73%	15.15%	29.60%	48.78%	
West	17.96%	9.10%	36.92%	56.24%	43.96%	15.91%	
**Disposition of patients**							
Routine	56.75%	60.07%	66.99%	64.47%	60.07%	68.23%	
Transfer to short-term hospitals	2.67%	2.81%	2.23%	2.83%	3.85%	1.98%	
Other transfers include SNF, ICF, etc.	16.74%	12.82%	9.33%	10.22%	15.59%	10.42%	
Home healthcare	18.51%	17.17%	16.08%	16.62%	15.06%	13.92%	
Length of stay (days)	6.52	8.09	7.467397	7.524489	6.922949	7.861343	<0.0001
Hospitalization charges (USD)	81,533.52	95,395.64	111,004.6	114,352.8	83,761.54	110,831.2	<0.0001

# Represents a quartile classification of the estimated median household income of residents within the patient’s zip code (https://www.hcup-us.ahrq.gov/db/vars/zipinc_qrtl/nrdnote.jsp, accessed on 1 March 2024). § The bed size cutoff points have been divided into small, medium, and large so that approximately one-third of the hospitals in a given region, location, and teaching status combination would fall within each bed size category (https://www.hcup-us.ahrq.gov/db/vars/hosp_bedsize/nrdnote.jsp, accessed on 1 March 2024). ~ A hospital is considered to be a teaching hospital if it has an American Medical Association-approved residency program (https://www.hcupus.ahrq.gov/db/vars/hosp_ur_teach/nrdnote.jsp, accessed on 1 March 2024).

**Table 2:** Comorbidities in patients of different racial groups with non-Hodgkin lymphoma in the United States from the NIS database from 2016 to 2019.

Comorbidities (Total = 777,740)	White (577,215)	Black (71,180)	Hispanic (73,000)	Asian/Pacific Islander (25,935)	Native American (2855)	Other (27,555)	*p*-Value
Hypertension	36.75%	36.68%	34.35%	34.37%	35.73%	35.73%	<0.0001
Diabetes mellitus	22.32%	26.43%	29.32%	26.89%	33.80%	23.15%	<0.0001
Smoking	6.96%	10.64%	4.41%	2.93%	12.43%	4.66%	<0.0001
Dyslipidemia	36.52%	25.44%	27.29%	32.64%	29.77%	26.84%	<0.0001
Obesity	9.95%	11.46%	10.40%	3.30%	11.73%	6.75%	<0.0001
Renal failure	20.45%	24.89%	18.00%	17.22%	20.49%	17.53%	<0.0001
Congestive heart failure	17.10%	16.82%	11.23%	11.20%	15.24%	10.56%	<0.0001
Chronic obstructive pulmonary disease	15.59%	10.96%	6.86%	6.11%	14.71%	7.78%	<0.0001
Pulmonary circulation disease	5.53%	6.31%	3.96%	3.49%	7.88%	3.90%	<0.0001
Coagulopathy	0.93%	1.26%	1.23%	1.39%	1.05%	1.00%	<0.0001
Depression	12.45%	7.76%	8.16%	4.51%	12.78%	8.13%	<0.0001

**Table 3:** Prevalence of MACCEs in patients of different racial groups with non-Hodgkin lymphoma in the United States from the NIS database from 2016 to 2019.

Outcomes (Total = 777,740)	White (577,215)	Black (71,180)	Hispanic (73,000)	Asian/Pacific Islander (25,935)	Native American (2855)	Other (27,555)	*p*-Value
Acute myocardial infarction	2.45%	1.79%	1.42%	1.83%	2.63%	1.07%	<0.0001
Atrial fibrillation	20.84%	10.18%	10.07%	12.92%	11.91%	12.52%	<0.0001
Cerebral events	4.29%	4.87%	3.64%	4.38%	4.55%	3.30%	<0.0001
Died during hospitalization	4.77%	5.64%	4.45%	5.51%	4.90%	4.81%	0.0002
Sudden cardiac death	0.67%	1.22%	0.77%	0.77%	0.88%	0.83%	<0.0001

**Table 4:** Adjusted odds ratios of major adverse cardiovascular and cerebrovascular events in patients of different racial groups with non-Hodgkin lymphoma in the United States from the NIS database from 2016 to 2019.

Race	Adjusted OR	95% Confidence Interval		Significance Value (*p*)
**All-cause mortality**				
White	Reference			
Black	1.276216	1.174679	1.38653	<0.001
Hispanic	1.069395	0.9814615	1.165207	0.125
Asian/Pacific Islander	1.277725	1.125053	1.451114	<0.001
Native American	1.134511	0.7686936	1.674418	0.525
Other	1.185762	1.014941	1.385333	0.032
**Acute myocardial infarction**				
White	Reference			
Black	0.7018127	0.6072979	0.8110371	<0.001
Hispanic	0.6900896	0.5908637	0.8059789	<0.001
Asian/Pacific Islander	0.880522	0.7105613	1.091136	0.245
Native American	1.14092	0.6940258	1.875577	0.603
Other	0.5749502	0.43748	0.7556178	<0.001
**Atrial fibrillation**				
White	Reference			
Black	0.6158417	0.5765031	0.6578647	<0.001
Hispanic	0.5728468	0.53643	0.6117359	<0.001
Asian/Pacific Islander	0.6982262	0.6289933	0.7750795	<0.001
Native American	0.6121325	0.4497586	0.8331272	0.002
Other	0.7705061	0.7047596	0.8423861	<0.001
**Cerebrovascular accident**				
White	Reference			
Black	1.000388	0.9084044	1.101686	0.994
Hispanic	0.9343409	0.8461065	1.031777	0.18
Asian/Pacific Islander	1.143281	0.9902107	1.320014	0.068
Native American	1.131307	0.7323143	1.747687	0.578
Other	0.9243223	0.7692416	1.110668	0.401
**Sudden cardiac death**				
White	Reference			
Black	1.817808	1.524071	2.168156	<0.001
Hispanic	1.220238	0.9926749	1.499967	0.059
Asian/Pacific Islander	1.232986	0.9005987	1.688049	0.191
Native American	1.358769	0.5607135	3.292687	0.497
Other	1.413797	0.9845441	2.030202	0.061

## Data Availability

The data utilized in this study is a publicly available resource under the Healthcare and Utilization Project of the Agency for Healthcare Research and Quality (AHRQ) in the United States. https://hcup-us.ahrq.gov/db/nation/nis/nisdbdocumentation.jsp.
